# Pathological Insights From Quantitative Susceptibility Mapping and Diffusion Tensor Imaging in Ice Hockey Players Pre and Post-concussion

**DOI:** 10.3389/fneur.2018.00575

**Published:** 2018-08-06

**Authors:** Alexander M. Weber, Anna Pukropski, Christian Kames, Michael Jarrett, Shiroy Dadachanji, Jack Taunton, David K. B. Li, Alexander Rauscher

**Affiliations:** ^1^Division of Neurology, Department of Pediatrics, University of British Columbia, Vancouver, BC, Canada; ^2^UBC MRI Research Centre, University of British Columbia, Vancouver, BC, Canada; ^3^Program of Cognitive Science, University of Osnabrueck, Osnabrueck, Germany; ^4^Department of Physics and Astronomy, University of British Columbia, Vancouver, BC, Canada; ^5^Division of Sports Medicine, Faculty of Medicine, University of British Columbia, Vancouver, BC, Canada; ^6^Department of Radiology, University of British Columbia, Vancouver, BC, Canada; ^7^MS/MRI Research Group, University of British Columbia, Vancouver, BC, Canada

**Keywords:** concussion, quantitative susceptibility mapping, diffusion tensor imaging, axial diffusivity, radial diffusivity, myelin, white matter, magnetic resonance imaging

## Abstract

Myelin sensitive MRI techniques, such as diffusion tensor imaging and myelin water imaging, have previously been used to reveal changes in myelin after sports-related concussions. What is not clear from these studies, however, is how myelin is affected: whether it becomes degraded and possibly removed, or whether the myelin sheath loosens and becomes “decompacted”. Previously, our team revealed myelin specific changes in ice hockey players 2 weeks post-concussion using myelin water imaging. In that study, 45 subjects underwent a pre-season baseline scan, 11 of which sustained a concussion during play and received follow-up scans: eight were scanned within 3 days, 10 were scanned at 14 days, and nine were scanned at 60 days. In the current retrospective analysis, we used quantitative susceptibility mapping, along with the diffusion tensor imaging measures axial diffusivity and radial diffusivity, to investigate this myelin disruption. If sports-related concussive hits lead to myelin fragmentation in regions of lowered MWF, this should result in a measurable increase in magnetic susceptibility, due to the anisotropic myelin fragmenting into isotropic myelin debris, and the diamagnetic myelin tissue being removed, while no such changes should be expected if the myelin sheath simply loosens and becomes decompacted. An increase in radial diffusivity would likewise reveal myelin fragmentation, as myelin sheaths block water diffusion out of the axon, with little to no changes expected for myelin sheath loosening. Statistical analysis of the same voxels-of-interest that were found to have reduced myelin water fraction 2 weeks post-concussion, revealed no statistically significant changes in magnetic susceptibility, axial diffusivity, or radial diffusivity at any time-point post-concussion. This suggests that myelin water fraction changes are likely due to a loosening of the myelin sheath structure, as opposed to fragmentation and removal of myelin debris.

## Introduction

Concussions are the most common form of traumatic brain damage, with between 1.6 and 3.8 million injuries per year in the United States alone (1). Despite how common they are, the underlying pathophysiological changes that take place after injury are poorly understood. One reason for this may be due to the lack of detectable changes by conventional magnetic resonance imaging (MRI). Current clinical neuroimaging techniques are unable to reliably detect, let alone quantify, signs of concussion, resulting in an inability to predict who will recover completely, who will have long-term impairments, or when it is safe to return to play in contact sports.

Recent advances in neuroimaging have provided more specific information on the sequelae of concussion, at least at a group level. Diffusion MRI, such as diffusion weighted imaging, diffusion tensor imaging, and diffusion kurtosis imaging, looks at the restriction of water diffusion to measure microstructural changes. These methods hold promise in traumatic brain injury (TBI) research due their sensitivity to microstructural changes in white matter (WM), such as axonal injury using axial diffusivity or myelin damage using radial diffusivity (2–4), but often times lack tissue specificity.

Myelin water imaging is another advanced MRI method, and is able to quantify metrics associated with changes specific to myelin, such as myelin water fraction (MWF) (5). In a prospective study in a cohort of 45 ice hockey players, we previously showed that the MWF is significantly reduced, upwards of 10% in some regions, 2 weeks after concussion compared to pre-injury baseline data, and then normalizes by 2 months after injury (6). Although myelin water imaging is more specific to myelin than any other MRI technique (7), it is not known from these data whether the observed changes in MWF are due to degradation/removal of myelin followed by establishment of a new myelin sheath, or due to a transient change in the structure of the myelin sheath, or a combination of both.

An MRI technique that may shed further light on these changes in myelin is quantitative susceptibility mapping (QSM). QSM is a relatively new technique that turns the resonance frequency measured with gradient echo MRI scans into maps of underlying tissue magnetic susceptibility (8). Strong modifiers of the magnetic susceptibility are the paramagnetic iron found in deoxygenated blood and in the basal ganglia, and diamagnetic myelin. In multiple sclerosis, significant magnetic susceptibility increases (MR frequency in earlier studies) have been seen occurring up to 3 months prior to lesion formation seen on gadolinium enhanced MRI (9). This increase in magnetic susceptibility was detectable by averaging across as few as 7 MS lesions. Due to its high sensitivity, QSM is widely used in MS research (10–12). The increase in MR frequency and magnetic susceptibility observed from myelin loss occurs due to anisotropic healthy WM degrading into more isotropic myelin debris, and the removal of diamagnetic myelin from the affected area (13). An important property of MR frequency and QSM is that its contrast to noise ratio is seven times higher than that of the corresponding magnitude (14). This high sensitivity to myelin degradation demonstrates that susceptibility sensitive MRI may allow for the distinction of decompaction—defined as the loosening of myelin sheaths around the axon and other myelin layers, with increased myelin water volume—from actual myelin breakdown.

One nice advantage to QSM analysis is that often times the required scan, SWI, is already routinely acquired when investigating concussion and TBI damage, due to its ability to detect and evaluate microhemorrhages (15). We had previously acquired susceptibility weighted images (SWI) in the same cohort of ice hockey players in which we performed myelin water imaging, and have previously reported finding no signs of microbleeds using this data (16). In recent years, QSM has undergone considerable maturation (17, 18), allowing us to now explore the magnetic properties of WM in this cohort.

DTI data, also acquired in the same study, can be used in tandem to corroborate these findings, as the DTI metrics previously mentioned, axial diffusivity (AD) and radial diffusivity (RD), have been shown to relate to axonal and myelin damage, respectively, in both animal (4, 19, 20) and human (3) models. In voxels of high anisotropy with aligned axons, AD is the measure of the primary eigenvalue, which is parallel to the axonal fibers. Damage to the axon can lead to reduced diffusion along this direction (4, 21, 22). Meanwhile, RD is the mean value of the secondary and tertiary eigenvalues, which run perpendicular to the fibers. Damage to myelin will lead to water diffusing perpendicularly out of the axon more easily, thus increasing RD (4, 19, 20).

Here, we investigated susceptibility changes along with axial and radial diffusivity in the same voxels-of-interest in which reductions of MWF were previously found (6) in 11 ice hockey players longitudinally after receiving a concussion during game-play. All players were scanned pre-season, and were subsequently scanned 3 days, 2 weeks, and 2 months post-injury. Based on animal studies that demonstrated decompaction of the myelin sheath after a single mild TBI (23), we hypothesized that the magnetic susceptibility and radial diffusivity would remain unchanged in areas of reduced MWF due to a lack of myelin fragmentation and removal.

## Methods

### Participants and data acquisition

Complete details of the original study have been previously reported (16). All subjects gave written informed consent prior to the study, which was approved by the University of British Columbia Clinical Research Ethics Board (H11-00423). Briefly, 20 female and 25 male ice-hockey players (mean age = 21.2 ± 3.1 years) underwent pre-season baseline and post-season clinical examination and MRI scans. If a player suffered a concussion during play, as diagnosed by a physician present during all games based on criteria outlined in the 3rd Consensus Statement on Concussion in Sport (signs of poor balance, confusion, and disorientation) (24), they were removed from play, given a clinical evaluation at the rink, followed by Sport Assessment Tool 2 (SCAT2) (24) tests in the dressing room. The players were then scheduled to receive additional follow-up scans at 72 h, 2 weeks, and 2 months following the concussion. All images were acquired with a 3T Philips Achieva scanner equipped with an 8-channel SENSE head coil. 11 players were concussed and eight were scanned within 72 h of being injured. 10 out of 11 athletes were scanned at 2 weeks, and nine out of 11 were scanned at 2 months post-injury.

All subjects underwent the following set of scans: (a) 3D-sagittal T1-weighted image (*TR* = 8.1 ms, *TE* = 3.7 ms, flip angle = 6°, voxel size = 1 × 1 × 1 mm^3^, acquisition matrix = 256 × 256 × 160, field of view = 256 × 256 × 160 mm^3^, SENSE factor = 2 along left-right direction); (b) DTI scan (TR/TE/flip angle = 7,015/60 ms/90°; acquisition matrix/field of view/acquired voxel size/reconstructed voxel size = 100 × 99/224 × 224 × 154 mm^3^/2.2 × 2.2 × 2.2 mm^3^/2 × 2 × 2.2 mm^3^; SENSE factor of 2.1 along the anterior-posterior direction, b_0_ = 0, b_1_ = 700 s/mm^2^, 60 non-collinear directions); and (c) multi-echo SWI with a 3D gradient echo (*TR* = 36 ms, *TE* = 6,12,18,24,30 ms, flip angle = 17°, acquisition matrix = 440 × 222 × 64, field of view = 220 × 166 × 128 mm^3^, voxel size = 0.5 × 0.5 × 1 mm^3^, SENSE factor = 1.2 along left-right direction).

Methods of the MWI acquisition and analysis can be found in Wright et al. (6) Briefly, a 32-echo T_2_-weighted scan was used to decompose the T_2_ decay using a non-negative least squares fit with an extended phase graph algorithm and flip angle optimization (25). MWF was calculated as T_2_ signal from 0 to 40 ms divided by the total T_2_ signal. MWF changes were evaluated through comparison of concussed athletes' baseline scans to those acquired at 72 h, 2 weeks, and 2 months post-injury. Voxelwise statistical analysis of the data was performed using tract-based spatial statistics (TBSS) (26) from the Functional MRI of the Brain Software Library (FSL, Oxford, United Kingdom) (27), created using fractional anisotropy maps obtained from diffusion tensor images.

### Post-processing

All multi-echo SWI images were post-processed as QSM images. For full details of this technique, please refer to Kames et al. (18). In brief, phase unwrapping was accomplished using a 3D Laplacian algorithm (28), while background field removal was performed by using the V-SHARP method (29). The inverse problem was solved using a two-step dipole inversion algorithm, first by addressing the well-conditioned k-space region by reconstructing using a Krylov subspace solver, and then reconstructing the ill-conditioned k-space region by solving a constrained l_1_-minimization problem (18). This proposed pipeline does not incorporate a priori information, but utilizes sparsity constraints in the second step. QSM was implemented using custom in-house Matlab code.

Raw diffusion data was first converted from Philips PAR/REC format to NIfTI using Chris Rorden's freely available dcm2nii (30) software (BSD License). Data was then eddy current and motion corrected using FSL's FDT (31) (FMRIB's Diffusion Toolbox) software. AD and RD values were calculated using the dcm2nii's calculated gradient directions and *b*-values and FSL's DTIFIT.

### Image analysis

**Figure 1:**
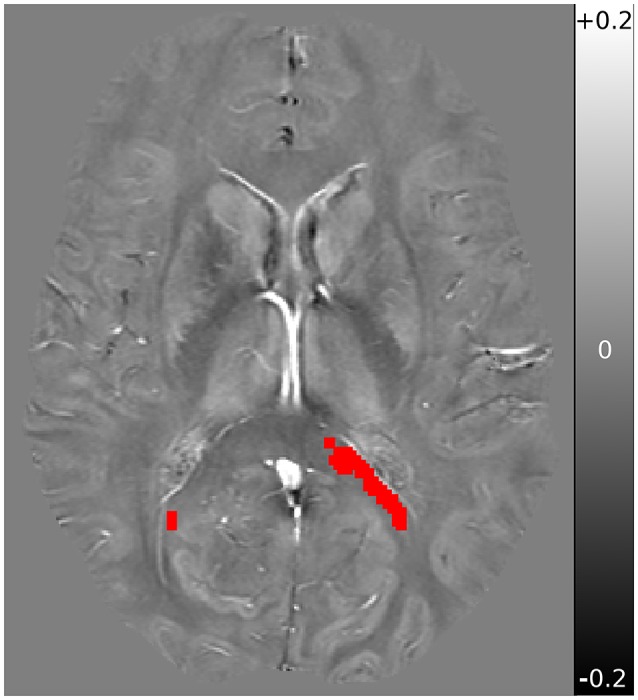
Sample QSM axial slice of a subject obtained at baseline, with overlay registered VOI mask in red. QSM underlay is shown in the range of −0.2 ppm (black) to +0.2 ppm (white), with 0 ppm shown in neutral gray.

FSL was further used for display, brain extraction, and registration of the voxels of interest. Registration between TBSS derived MWF significant voxels and QSM/DTI was accomplished using FSL's FLIRT (32), and were inspected individually. The mean QSM, AD, and RD values from within the previously identified voxels of interest (VOI) from Wright et al. (6) were then computed. A sample QSM axial slice image, with VOI overlayed, is shown in Figure 1.

### Statistics

Statistical analysis tools R (33), lme4 (34), and languageR (35) were used to perform a linear mixed-effects analysis on the relationship between QSM, AD and RD values and time. Fixed effects were set as gender and age, while random effects were set for subjects, including by-subject random slopes for the effect of time:

$$\begin{array}{l} \left[\text{QSM}/\text{AD}/\text{orRD}\right]\sim \text{Time}+\left(1|\text{Subject}\right)+\text{Age}+\text{Sex}+\varepsilon \end{array}$$

Visual inspection of residual plots did not reveal any obvious deviations from homoscedasticity or normality. A linear mixed-effects model was used due to its advantages in dealing with missing data (36). Time was treated as a numeric variable. *P*-values were obtained by likelihood ratio tests of the full model with time against the “null” model without time, with a *p*-value of 0.05 set as the threshold required to reject the null hypothesis. Power was calculated for the QSM retrospective analysis using 10,000 simulated longitudinal studies of similar size using baseline mean and standard deviation values from our results and expected QSM changes to calculate effect size. A further 10,000 power simulations were performed resulting in a power estimate of 88% (see Supplementary Materials).

## Results

**Figure 2:**
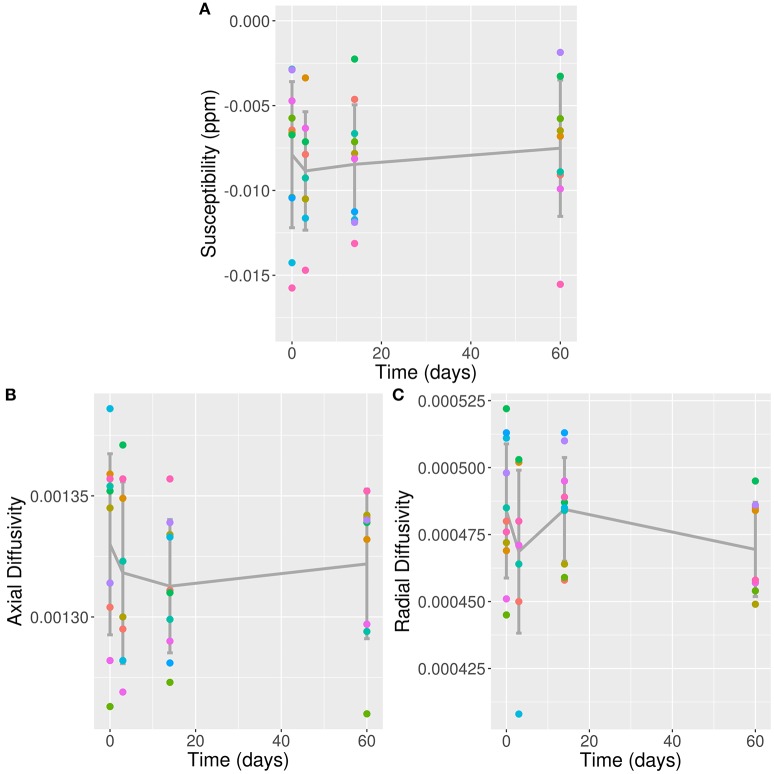
QSM, AD, and RD Values in VOI Over Time. **(A)** QSM values (in ppm); **(B)** AD values; and **(C)** RD values plotted against time post-concussion by individual subjects separated by color, and mean and standard deviation (error bars) plotted in dark gray; against time post-con Note: time zero refers to baseline scan.

All 11 concussed subjects [five male, mean age 21.18 ± 1.66 (SD) years] scored 15 on the Glasgow Coma Scale, indicating a mild TBI/concussion. They were all scanned during the preseason (baseline), with eight participating in the 3 day follow-up scan, 10 at 2 weeks, and nine at 2 months. Results from conventional MRI, MWI, and psychometrics were reported previously (6, 16). Previous results of particular relevance to the present paper are a cluster of voxels detected using TBSS with significantly reduced MWF at 2 weeks post-concussion; no other time-points achieved statistical significant changes (6). The voxel clusters were located in the splenium of the corpus callosum, right posterior thalamic radiation, left superior corona radiata, left superior longitudinal fasciculus, and left posterior limb of the internal capsule. Across all significant voxels, this represented a 5.9 ± 1.2% (mean ± standard error) decrease from baseline, and upwards of 10% reduction in voxels located in the left splenium. Mean QSM values from all 11 concussed subjects at baseline in the VOI was −0.0079 ppm (confidence interval: −0.0104 to −0.0053). Longitudinal values are listed in Table 1. The fixed effects of gender and age did not show any statistically significant influence on the model. Likelihood ratio-test analysis of the full model with time against the “null” model without time did not show a significant change in QSM values in the VOI (*p* = 0.94; Figure 2A).

**Table 1:** Mean QSM, AD, and RD values with standard deviations and confidence intervals.

		**Baseline**	**72 h**	**2 Weeks**	**2 Months**	***p*-Value**
	***n* =**	**11**	**8**	**10**	**9**	
QSM	Mean SD CIs	−0.0079 0.0043 −0.0104 to −0.0053	−0.0089 0.0035 −0.0112 to −0.0064	−0.0085 0.0035 −0.0106 to −0.0063	−0.0075 0.0040 −0.0101 to −0.0049	0.94
AD	Mean SD CIs	0.0013 3.7e-5 0.0013 to 0.0014	0.0013 3.8e-5 0.0013 to 0.0013	0.0013 2.8e-5 0.0013 to 0.0013	0.0013 3.1e-5 0.0013 to 0.0013	0.92
RD	Mean SD CIs	0.00048 3.0e-5 0.00047 to 0.00050	0.00047 3.0e-5 0.00045 to 0.00049	0.00049 1.9e-5 0.00047 to 0.00050	0.00046 1.7e-5 0.00046 to 0.00048	0.25

*Mean values over time in the VOI, with p-values from likelihood ratio test. n, number of subjects scanned at time-point; SD, standard deviation; CI, confidence interval*.

Mean AD and RD values from all 11 concussed subjects over time are listed in Table 1. The fixed effects of gender and age did not show any statistically significant influence on either model. Likelihood ratio-test analysis of the full model with time against the “null” model without time did not show a significant change in AD or RD values in the VOI (*p* = 0.92 and 0.25, respectively; Figures 2B,C).

## Discussion

This study used measurements of WM magnetic susceptibility along with DTI measures AD and RD to examine the impacts of concussion on the brain of varsity ice hockey players. To our knowledge, this is the first study to look for changes in magnetic susceptibility, from data before injury, and at 3 days, 2 weeks, and 2 months post-injury. Our data revealed no statistically significant QSM, AD or RD changes at any time-point post-concussion. This finding has implications for how myelin is affected by a concussive hit in the first 2 months post injury.

In the same cohort of patients as in the present study, we have previously reported finding a significant reduction in MWF, with up to 10% reduction seen in the sCC (6). At the time, however, we could not deduce what was causing this MWF reduction: myelin degeneration, myelin sheath loosening (decompaction), or a mix of the two. A previous study by Johnson *et al*. provided evidence for myelin degeneration and active phagocytosis of myelin fragments in humans following moderate/severe TBI (37). Another study by Donovan et al. demonstrated that repeated mTBI in rats leads to a spectrum of changes, including separation of the myelin sheath from the axon, decompaction of the myelin sheath, and fragmentation of the myelin sheath (23). Finally, investigations into secondary degeneration in rat optic nerves, characterizing ongoing changes associated with neurotrauma, have shown that myelin is particularly susceptible to secondary damage, which can lead to myelin sheaths becoming loose (38, 39). Payne et al. found a maximum of 15% of myelin sheaths to be decompacted in rats following secondary degeneration (39). This is due to the fact that myelin's compact layers of lamellae are held together with proteins that are vulnerable to damage from reactive oxidative species and lipid peroxidation from secondary degeneration (40), processes we know occur following concussive hits and mTBI (41, 42). Thus, there is circumstantial evidence to support myelin decompaction, a mixture of decompaction and degeneration, or only degeneration, following a concussion.

Together, the reduction in MWF, and the absence of statistically significant changes in magnetic susceptibility or RD in the same region observed in the current study, suggest that the myelin sheath structure has been altered, such as becoming decompacted, rather than degraded or fragmented (23, 39). Degradation or removal of myelin should result in an increase in magnetic susceptibility and an increase in radial diffusivity. The decompaction interpretation is in agreement with the observed recovery of the myelin water fraction by 2 months post-injury, suggesting a normalization of the myelin sheath structure.

Decompaction, while not as severe as myelin degeneration, should still be considered a serious injury, as it leads to reductions in action potential conduction (43). Unmyelinated axons, in mice and rats, have a conduction rate of 0.4 m/s, significantly lower than the myelinated conduction rate of 2.4 m/s (44, 45). Axons of mice with decompacted myelin, however, have a conduction rate of about 1.05 m/s, a reduction of more than half the healthy rate (43). This reduction in conduction could be responsible for some of the known cognitive deficits following concussive hits (46), such as affected memory, attention, processing speed, and executive functioning.

Myelin decompaction is likely to be caused by secondary mechanisms, such as oxidative stress (39). Petronilho et al. looking at oxidative damage following mTBI and severe TBI in adult male Wistar rats, found an inversely proportional link between trauma severity and oxidative damage (47). Thus, for mTBI, there was more evidence of oxidative stress than in the severe TBI rats. What secondary mechanisms could be causing this separation of the myelin layers? While iron is a known reactive oxidative species, and has been implicated in mTBI secondary damage (41), we would expect an increase in magnetic susceptibility if iron levels were increased, for example due to hemorrhage related formation of hemosiderin. As reported, no such increase in susceptibility was observed. Other potential candidates include high levels of radical species such as nitric oxide and hydroxyl radicals (39). Ultimately it is beyond the scope of this paper to identify the exact cause of this decompaction.

One possible criticism of our interpretation of the previously reported MWF results is that MWF changes can occur due to increased edema/inflammation instead of changes to the myelin layers. While it is true, in theory, that MWF can be modified by edema/inflammation (48), this is likely not the case in our present study. As demonstrated previously by Chiang et al., an increase in extracellular fluid, such as vasogenic edema, should lead to increased radial diffusivity, and a partial increase in axial diffusivity (49). Neither AD or RD showed increases in the same regions that showed reduced MWF as previously reported. If edema was to blame for this reduction in MWF, we should expect a simultaneous increase in AD and RD, which is not seen. Furthermore, as demonstrated in our 2016 Frontiers in Neurology publication, no microbleeds or hemorrhages were detected as a result of concussion or playing a season of ice hockey, nor was there any increase in brain volume (16). Finally, as stated in our 2016 PLOS One publication, we found decreases in MWF of up to 10% in voxel clusters in the sCC (6). For “diffuse edema” to explain this change, we would likely see a reduced MWF throughout the whole brain (which was not observed), and a ~9% swelling of the brain, which would cause enough intracranial pressure to prove lethal (50).

Another counter to our proposed explanation would be to suggest that perhaps myelin degradation is occurring, but that the increased magnetic susceptibility is being masked by a concurrent and equal reduction in susceptibility due to some other factor. This, however, is negated by our finding no changes in AD or RD over time, suggesting that axial damage and myelin fragmentation is not occurring. Furthermore, in order for QSM values to remain constant despite myelin loss, an equivalent reduction in iron should be expected. A reduction in iron, however, is highly unlikely given the past literature (see Nisenbaum et al.'s review of iron in mTBI in the Journal of Neurotrauma) (41).

There are several limitations to this work that should be highlighted and addressed in future studies. This study included some missing data points of subjects. A linear mixed-effects model was therefore used due to its ability to handle missing data-points. Another limitation is our decision to only look at the region where myelin water imaging detected a reduction in myelin signal in the same cohort. Since myelin water comprises only about 10% of tissue water, MWI is a noisy technique by definition. It is possible that other areas were damaged but not detected by MWI.

Finally, we do not know what may happen between 2 weeks and 2 months after concussion, and after 2 months post-concussion. Future studies should have an MRI scan at 4 weeks, 6 months and up to 1 year post injury to provide additional information on the trajectory of recovery after injury. In particular, denser sampling of the time period between 2 weeks and 2 months after concussion will provide further insight on the time course of tissue recovery after concussion. Such work will lead to a better understanding of how much later tissue recovery succeeds functional recovery. Such knowledge is critical for return to play decision making in contact sports, which is based on clinical assessment of functional recovery.

In summary, we report a repeated measures QSM, AD and RD analysis of the same regions previously reported to have reduced MWF due to sports related concussion. We did not find any statistically significant changes in magnetic susceptibility, axial diffusivity, or radial diffusivity in these regions after 3 days, 2 weeks, or 2 months post-concussion. This finding provides evidence that a sports-related concussion leads to decompaction in myelin sheaths, as opposed to myelin degradation.

## Data and materials availability

Data is available and may be provided under the transfer policies of UBC.

## Disclosure

DL has received research funding from the Canadian Institute of Health Research and Multiple Sclerosis Society of Canada. He is the Emeritus Director of the UBC MS/MRI Research Group which has been contracted to perform central analysis of MRI scans for therapeutic trials with Novartis, Perceptives, Roche and Sanofi-Aventis. The UBC MS/MRI Research Group has also received grant support for investigator-initiated independent studies from Genzyme, Merck-Serono, Novartis and Roche. He has acted as a consultant to Vertex Pharmaceuticals and served on the Data and Safety Advisory Board for Opexa Therapeutics and Scientific Advisory Boards for Adelphi Group, Celgene, Novartis, and Roche. He has also given lectures which have been supported by non-restricted education grants from Teva, Novartis and Biogen.

## Author contributions

AR, JT, and DL designed the study. AR and DL designed the imaging protocol. SD collected data and helped coordinate the study. CK wrote the QSM algorithm. AP performed initial data and statistical analysis under the supervision of AW. AW performed subsequent data and statistical analysis. AP wrote the initial manuscript with major revisions by AW. MJ performed initial DTI analysis. AR and MJ edited and provided critical input to the manuscript. All authors interpreted the data. All authors had full access to the data, and helped critically revise the manuscript before reviewing and approving the final version.

## Conflict of interest statement

The authors declare that the research was conducted in the absence of any commercial or financial relationships that could be construed as a potential conflict of interest.
