# Supplementary material for: Pathological Insights From Quantitative Susceptibility Mapping and Diffusion Tensor Imaging in Ice Hockey Players Pre and Post-concussion
Source: Front Neurol. 2018 Aug 6;9:575. doi: 10.3389/fneur.2018.00575 (PMC6091605; doi:10.3389/fneur.2018.00575)
Supplement: Supplementary file 1 [file Data_Sheet_1.DOCX]

Supplementary Materials


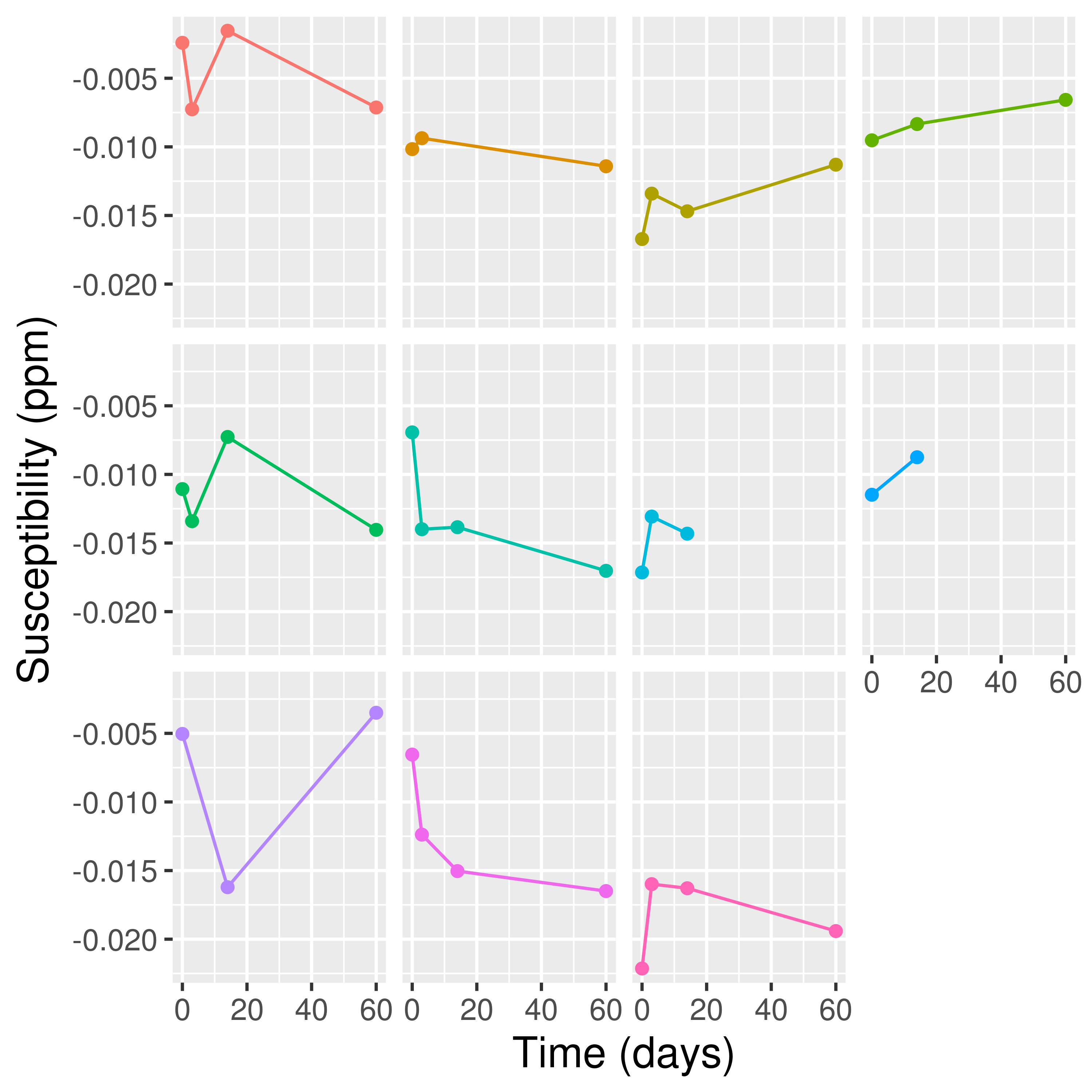
Additional Figures:

**Supplementary Figure 1: Individual Line and Dot Plots of QSM Values in sCC VOI Over Time.** Raw QSM values (in ppm) plotted against time by individual subjects.

Statistical Assumption Analysis:

*Normality:*


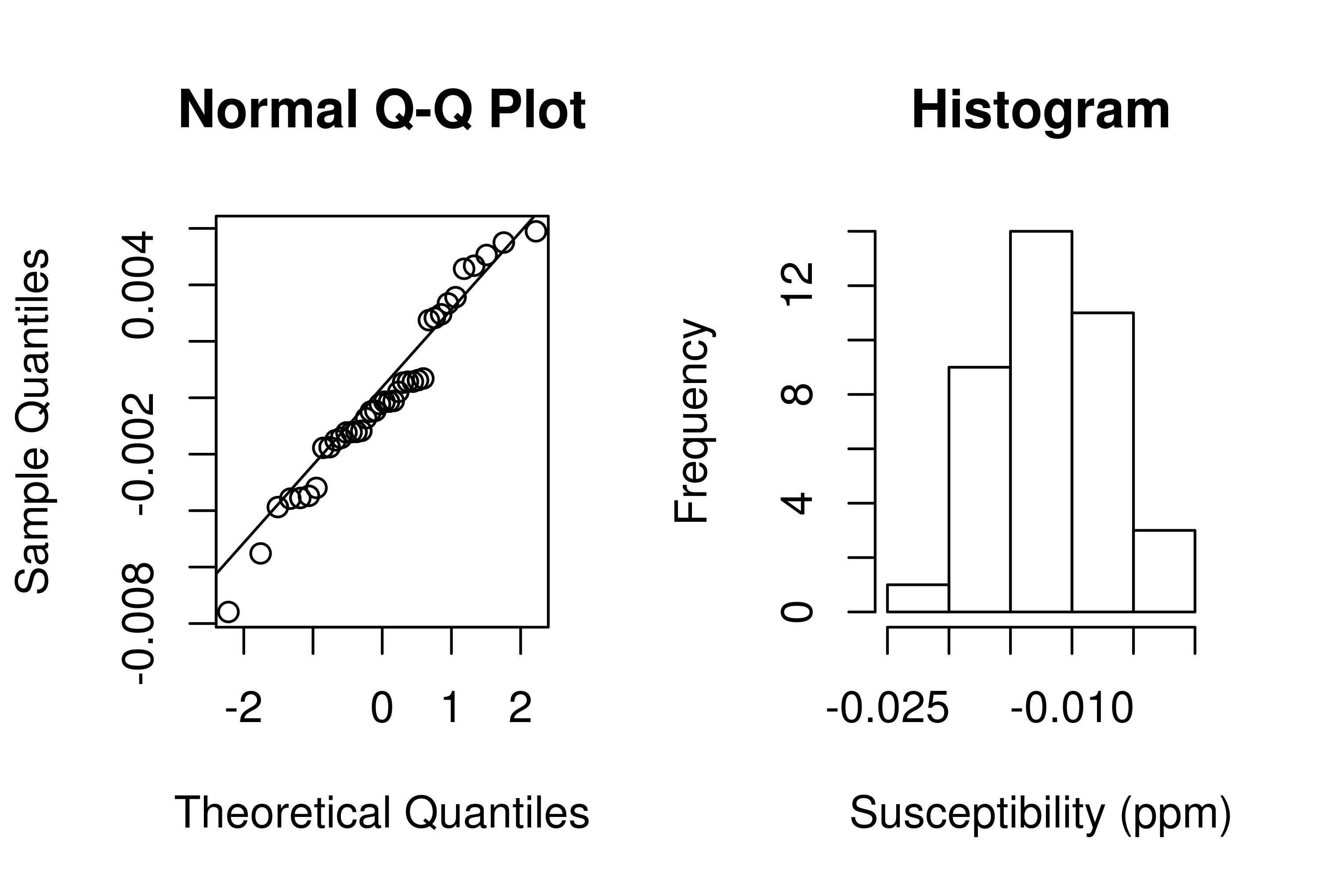
*Linearity and Homoskedasticity:*


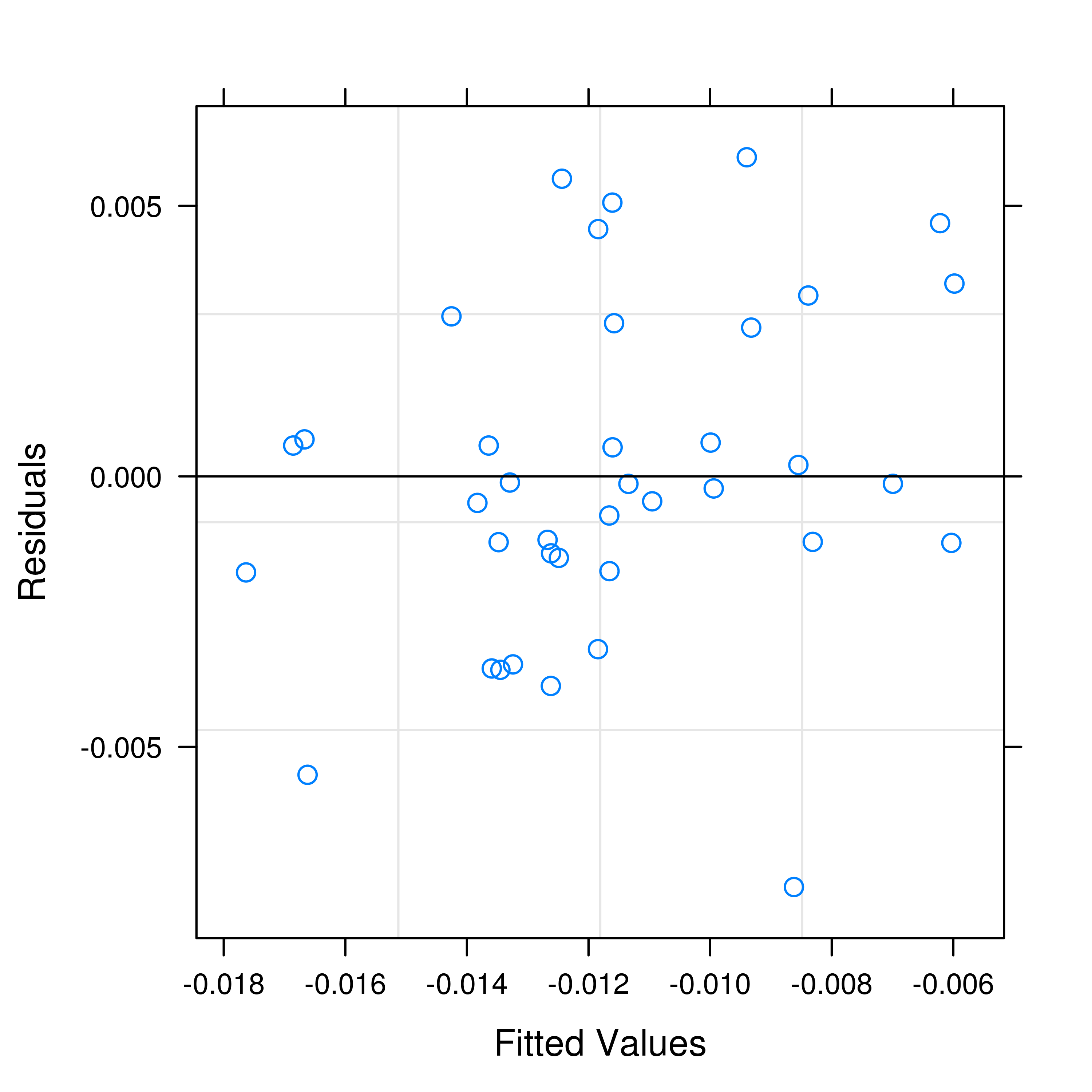


Power Calculations:

Power was calculated using simulated data of the minimum effect size we expected to see given a 10% reduction in MWF at two weeks post injury.

In order to determine the amount of QSM change we could expect if myelin was degraded and removed, we compared our findings to those in multiple sclerosis (MS), where myelin degeneration has been reported using both phased-based MRI and magnetization transfer (MT). MT was used as there are no directly comparisons of MWI with phase-based MRI findings. MT and MWF both provide indirect measures of myelin, with MTR to myelin staining R^2^ values previously reported between 0.2 and 0.71, and MWF to myelin staining R^2^ values reported between 0.56 and 0.67.^1^

Filippi et al., looking at serial monthly MT scans in MS patients, found a 10% reduction in the MT ratio (MTR) three months before gadolinium (Gd) enhancing MS lesions appeared in the same region.^2^ This can be compared to MR frequency shift increase (a phase based MRI method similar to QSM) findings in MS at three months before Gd enhancing lesions, as found in Wiggerman et al.'s 2013 Neurology paper, in which they report an increase of 0.0014 ppm.^3^

^
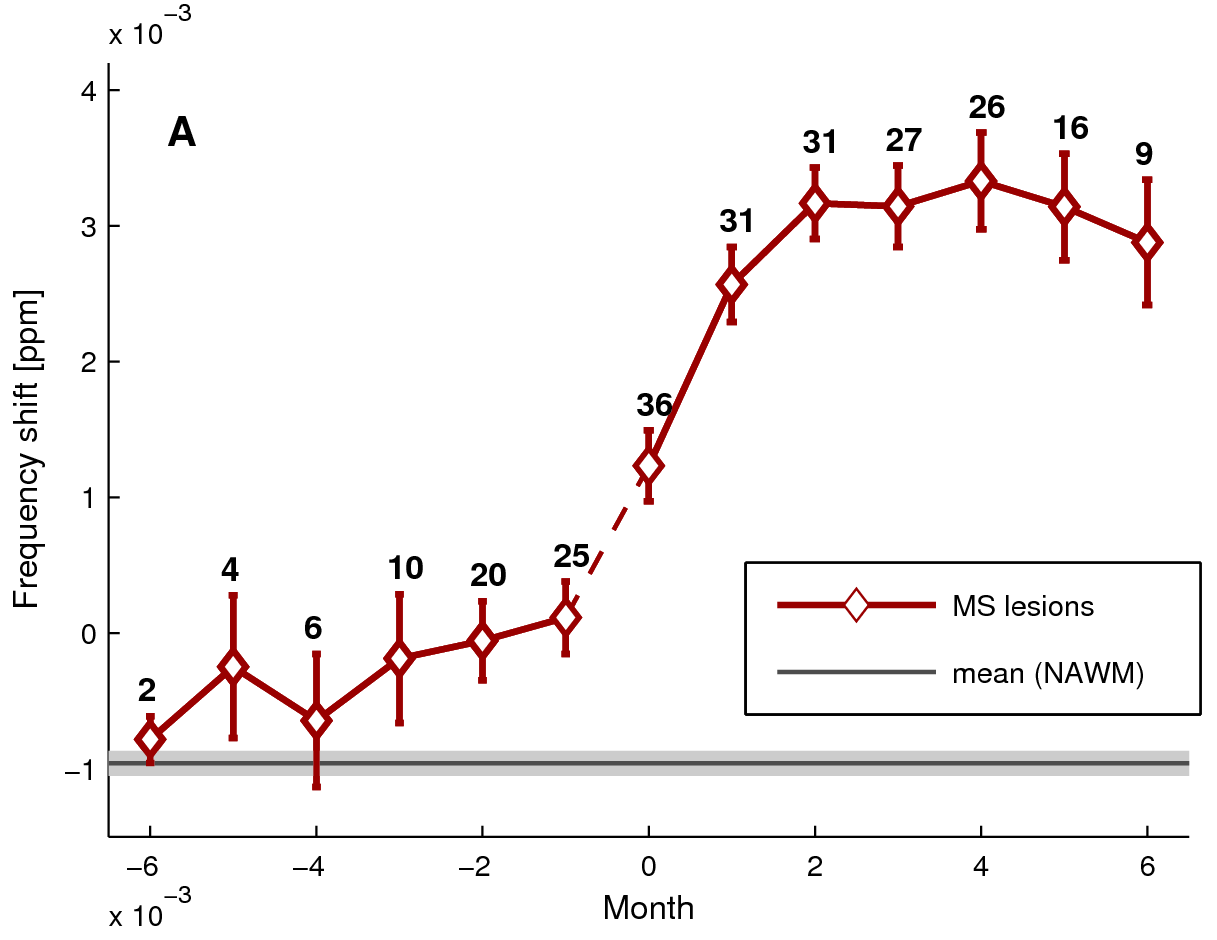
^

S**upplementary Figure** **2** (for review only): Changes in MR frequency due to MS lesion formation in 20 patients (Wiggermann et al. Neurology 2013). Month 0 is the time when a lesion first appears on MRI as an enhancement with Gadolinium. Data were available from six months prior to and six months after enhancement. The numbers along the curve indicate the number of lesions evaluated. The error bars indicate standard error of the mean. NAWM = normal appearing white matter. The most notable feature of this curve are (1) that three months prior to lesion appearance, there is a significant increase in MR frequency and (2) that demyelination causes very strong increases in frequency, as seen from two months after enhancement onward. The gray shaded area around the mean of NAWM indicates the standard error in NAWM, which is indicative of the high reproducibility of the method over several scans.

Thus, with a 10% decrease in MWF, as found in our 2016 MWI report,^4^ we would expect a 0.0014 ppm increase in magnetic susceptibility for an effect size, if the change in MWI was due to removal of myelin.

Using baseline mean and standard deviation values from our QSM results, we simulated^5^ 10,000 longitudinal studies with 10 subjects with an increase in magnetic susceptibility of 0.0014 ppm at two weeks. An increase of 0.0007 ppm was also included at the three day mark, as per Wright et al.'s findings, and a return to baseline at two months.

Performing the same linear mixed-effects model and likely ratio test calculations on all 10,000 simulated data sets resulted in a effect size of 7.5e-05. Using this effect size and an alpha value of 0.05, 10,000 power simulations were performed on our own likelihood ratio test, resulting in a power of ~88%:

Power for predictor 'Time', (95% confidence interval):

87.63% (86.97, 88.27)

Test: Likelihood ratio

Effect size for Time is 7.5e-05

Based on 10000 simulations, (3 warnings, 0 errors)

alpha = 0.05, nrow = 38

References:

1. Laule, C. *et al.* Magnetic resonance imaging of myelin. *Neurother. J. Am. Soc. Exp. Neurother.* **4,** 460–484 (2007).

2. Filippi, M., Rocca, M. A., Martino, G., Horsfield, M. A. & Comi, G. Magnetization transfer changes in the normal appearing white matter precede the appearance of enhancing lesions in patients with multiple sclerosis. *Ann. Neurol.* **43,** 809–814 (1998).

3. Wiggermann, V. *et al.* Magnetic resonance frequency shifts during acute MS lesion formation. *Neurology* **81,** 211–218 (2013).

4. Wright, A. D. *et al.* Myelin Water Fraction Is Transiently Reduced after a Single Mild Traumatic Brain Injury - A Prospective Cohort Study in Collegiate Hockey Players. *PloS One* **11,** e0150215 (2016).

5. Goldfield, K. *simstudy: Simulation of Study Data*. (2017).
